# Editoral Brevities

**Published:** 1888-05

**Authors:** 


### EDITORIALS AND MISCELLANEOUS.

### EDITORIAL BREVITIES.

GELSEMIUM FOR RIGID OS —The *Medical Times* mentions that Dr. Freeman recommends gelsemium to relieve the irregular pains which precede labor and to relax a rigid os. This is nothing new in this section of country.

FAIRCHILD BROS. & FOSTER. This excellent house made a fine exhibit of their goods at Georgia Medical Association in Rome, on the 18th ult. Their pepsin preparations are of a superior kind. See their advertisement.

MESSRS. REED & CARNRICK had a beautiful disp'ay of their goods and specialties at the late meeting of the Medical Association in Ro ne. This is a good house, and their preparations are of a superior order. See their advertisement in this journal.

SEABURRY & JOHNSON.—Whose advertisement may be seen in this Journal had a splendid display of their goods at the late meeting of the Georgia Medical Association. This is an excellent and long established house. They are well represented by their agent, Dr. A. H Beck.

I. H. BATES, the well known advertising agent in New York city, has changed his place of business from No. 41 to No. 38 Park Row, New York. We have been doing business with Mr. Bates for a number of years, and have always found him a prompt and reliable business man.

I. PHILLIPS, the instrument man of Atlanta, had a fine display of surgical instruments, drug cases, etc., etc. at the late meeting of the Georgia Medical Association, at Rome. Phillips is a safe, polite and active business man. He always keeps an advertisement in our Journal. Read it.

MCARTHUR'S HYPOPHOSPHITES comes to us highly recommended, especially in lung affections and in low states of the system. This preparation stands deservedly high, and we commend it to the profession. See the advertisement of McArthur's Hypophosphite Co. on page opposite third cover.

CENTINARIAN PROFESSORS.—The French take the palm for longevity among the teachers in medicine. Professor *Chevrul* is in the French Institute of Sciences at Paris, and Professor *Neuron* is Professor of Anatomy at College of Notterdam, Ind., both have passed their hundredth year.

HYPODERMIC TABLETS.—We have received samples of the hypodermic tablets from the house of Parke, Davis & Co., Detroit, Michigan. They are beautiful and evidently prepared with great care and accuracy. They have also prepared improved nickel plated hypodermic cases. These tablets will prove a great convenience to the profession, and the new cases will, we think, soon be in general demand by practitioners.

CHANGE OF THE DRACHM (ʒ) MARK.—The journals are discussing the change of the present drachm mark to the greek letter delta △. It has been objected that this would be as likely to cause mistakes, because difficult to make. We think not. It is but a triangle, and is certainly as easy to make as anything else that can be adopted, and as little likely to cause mistakes. Let us adopt it.

VISIT OF MEDICAL FRIENDS.—Since our last issue we have had the pleasure of calls from the following medical brethren, all good and true men in the profession: Dr. P. L. Cortelvou, of Marietta, Ga ; Dr. L. L. Clement, of Mazeppa, Ga.; Dr. A. T. Parke, of Walker county, Ga.; and Dr. Gann, of Bolton, Georgia.

DISPLAYS OF CHEMICALS.—The Mellier Drug Co., importers of and dealers in surgical instruments, and manufacturers of saddle bags, and that excellent preparation tongaline, were present at the Georgia Medical Association in the person of their polite agent. Mr Jack Frost. This well known establishment has an advertisement in this Journal, which see.

BEAUTIFUL CHEMICAL PREPARATION.—We learn that a snow white mass of caffeine, the active principle of coffee, (200 pounds and of great value,) is now on exhibition in the window of Wm. R. Warner & Co., 1228 Market street. This beautiful crystallization represents ten tons of coffee, and is used as an ingredient in the preparation of bromo soda prescribed for the cure of headeaches, migraine, nervousness and sea sickness.

